# Do clinically anxious children cluster according to their expression of factors that maintain child anxiety?

**DOI:** 10.1016/j.jad.2017.12.078

**Published:** 2018-03-15

**Authors:** Samantha Pearcey, Anna Alkozei, Bhismadev Chakrabarti, Helen Dodd, Kou Murayama, Suzannah Stuijfzand, Cathy Creswell

**Affiliations:** a School of Psychology and Clinical Language Sciences, University of Reading, PO Box 238, Reading RG6 6AL, United Kingdom; b Department of Psychiatry, University of Arizona, 1501 N. Campbell Ave, Tucson, AZ 85721, United States

**Keywords:** Anxiety, Children, Treatment, CBT, LPA

## Abstract

**Background:**

Cognitive Behaviour Therapy (CBT) is an effective treatment for childhood anxiety disorders, yet a significant proportion of children do not benefit from it. CBT for child anxiety disorders typically includes a range of strategies that may not all be applicable for all affected children. This study explored whether there are distinct subgroups of children with anxiety disorders who are characterized by their responses to measures of the key mechanisms that are targeted in CBT (i.e. interpretation bias, perceived control, avoidance, physiological arousal, and social communication).

**Methods:**

379 clinically anxious children (7–12 years) provided indices of threat interpretation, perceived control, expected negative emotions and avoidance and measures of heart rate recovery following a speech task. Parents also reported on their children's social communication difficulties using the Social Communication Questionnaire (SCQ).

**Results:**

Latent profile analysis identified three groups, reflecting (i) ‘Typically anxious’ (the majority of the sample and more likely to have Generalized anxiety disorder); (ii) ‘social difficulties’ (characterized by high SCQ and more likely to have social anxiety disorder and be male); (iii) ‘Avoidant’ (characterized by low threat interpretation but high avoidance and low perceived control).

**Limitations:**

Some measures may have been influenced by confounding variables (e.g. physical variability in heart rate recovery). Sample characteristics of the group may limit the generalizability of the results.

**Conclusions:**

Clinically anxious children appear to fall in to subgroups that might benefit from more targeted treatments that focus on specific maintenance factors. Treatment studies are now required to establish whether this approach would lead to more effective and efficient treatments.

## Highlights

• Latent profile analysis suggested clinically anxious children represented three groups.
• The groups differed on putative maintenance mechanisms often targeted in CBT.
• The groups were categorised as “Typically anxious”, “Social Difficulties” and “Avoidant”.
• Groups did not neatly align with existing diagnostic categories for child anxiety disorders.

## 1 Introduction

Anxiety disorders affect approximately 6.5% of children worldwide (Polanczyk et al., 2015). The mean age of onset is 11 years of age (Kessler et al., 2005) yet anxiety disorders often persist into adulthood (Kessler et al., 2005) and increase the risk of other psychopathologies throughout life (Bittner et al., 2007). The high prevalence, persistence and impairment associated with childhood anxiety disorders highlights the need for effective interventions.

Currently, the recommended first line treatment for anxiety in pre-adolescents is typically a general form of Cognitive Behavioral Therapy (CBT) that can be applied across a range of anxiety diagnoses (e.g. Kendall, 1994; Kendall et al., 2002; Pilling et al., 2013). Such general forms of CBT typically target mechanisms that appear in adult maintenance models of anxiety (e.g. Rapee and Heimberg, 1997), which are thought to also play a role in maintaining anxiety in children, including negative thinking styles (in particular threatening interpretations of ambiguous information and low self-efficacy), abnormal physiological arousal, avoidance of feared stimuli and, in some cases, social communication deficits (Albano and Kendall, 2009, Rapee et al., 2000).

The effectiveness of general CBT is promising (59.4% recovery) when compared to waitlist controls (17.5%; James et al., 2013) and there are fewer side effects when compared to pharmacotherapy (Rynn et al., 2015). However, almost half of the children who receive CBT retain a diagnosis and, as such, there is clear room for improvement.

In order to improve treatment for children with anxiety disorders, it is necessary to understand the reasons why they are not effective for some children. A number of demographic and clinical characteristics have previously been associated with impaired outcomes (i.e. higher symptom severity, lower socio-economic status (SES) and comorbid diagnoses of other anxiety, mood and behavioral disorders; Compton et al., 2014; Hudson et al., 2015). However, it may also be the case that the mechanisms that are targeted in general forms of CBT are not appropriate for all anxious children. Indeed, the evidence for the presence of these mechanisms in childhood anxiety disorders is unclear. For example, negative thinking styles have not consistently been found amongst anxious youth in comparison to non-anxious youth, particularly when samples are restricted to pre-adolescents (Waite et al., 2015, Waters et al., 2008). Furthermore, although avoidance of feared stimuli is often associated with anxiety in children (Lebowitz, 2017) it is not always required for a diagnosis (e.g. in social anxiety disorder where enduring with distress may be an alternative to avoidance; DSM-5, 2013)

Whilst there is some evidence that children with anxiety disorders, compared to non-anxious children, show reduced heart rate (HR) recovery following a stressor (Schmitz et al., 2011, Schmitz et al., 2013) others have found no, or only marginal differences (e.g. Alkozei et al., 2015; Beidel, 1991). When it comes to social communication deficits, there is some evidence for both self and observer rated social communication deficits in groups of children with both mixed anxiety disorders (e.g. Dodd et al., 2011) and social anxiety disorder specifically (Spence et al., 1999) compared to non-anxious children. However, others have only found evidence for deficits according to self-, but not observer-ratings (e.g. Cartwright-Hatton et al., 2003, Cartwright-Hatton et al., 2005). These mixed findings are complicated by the potential for anxiety-driven inhibited behaviors in social situations to be coded as skills deficits (e.g. poor eye contact) in observational studies. Although, notably, recent studies of underlying social communication deficits have indicated that children with anxiety disorders are more likely than non-anxious children to display social communication difficulties (van Steensel et al., 2013).

The inconsistencies that have been found across studies may reflect the presence of subgroups of children for whom these maintenance mechanisms apply to different degrees. Given that many of these studies include samples of children with a variety of anxiety disorders, it is possible that these subgroups represent different diagnostic categories. However, to date there has been little evidence for diagnostic specificity in relation to negative thinking (Creswell et al., 2014) and physiological arousal (Alkozei et al., 2015) although there is some evidence that social communication difficulties may be more common among children with social anxiety disorder than other anxiety disorders (Halls et al., 2015). These findings suggest that, in order to deliver treatments that optimize outcomes, children with anxiety disorders may be better categorized according to the presence of particular maintenance mechanisms than by traditional diagnostic categories.

As such, and in line with the precision psychiatry approach that uses data driven techniques to identify subgroups within standard psychiatric categories (Fernandes et al., 2017), the current paper uses a person centered mixed models approach (Latent Profile Analysis; LPA) to explore the following research questions; (i) are there distinct subgroups of clinically anxious children that differ in their expression of the core maintenance mechanisms that are targeted in CBT (i.e. negative thinking styles (interpretation bias, expected negative emotions and expected control), avoidance, physiological arousal and social communication difficulties)?; (ii) do these subgroups align with existing diagnostic categories for anxiety disorders in children?; and (iii) do these subgroups differ on clinical characteristics that commonly predict treatment outcome (i.e. symptom severity, SES and the presence of SoAD, mood and behavioral disorders)?

## 2 Method

### 2.1 Participants

Four hundred and six clinically anxious children were recruited to one of two treatment trials (Creswell et al., 2015, Thirlwall et al., 2013) through the local child and adolescent mental health service (CAMHS) following referral by local health and education professionals. The children included in these trials were aged 7–12 years, met criteria for a primary anxiety disorder diagnosis, did not have a significant physical or intellectual impairment (including autism spectrum disorders), were not currently prescribed psychotropic medication, and their primary carer did not have a significant intellectual impairment (that would have inhibited participation in subsequent treatment). Research assessments were carried out prior to the commencement of any treatment.

The current analyses included 379 participants (see Table 1). Children who were excluded (*N* = 27) on the basis of having data for none (*n* = 5) or only one (*n* = 16) of the dependent variables, or being outside of the study age range at the time of assessment (*n* = 6), did not differ significantly from the included sample on age (Welch's *F*(1, 10.17 = .005, *p* = .95), gender (*χ*^*2*^ (1) = .15, *p* = .70) or primary diagnosis CSR (Welch's *F*(1, 10.44) = 2.56, *p* = .14). Compared to non-participants, participants were less likely to have a primary diagnosis of Specific Phobia (*χ*^*2*^(1) = 6.75, *p* = .01).

**Table 1:** Sample characteristics.

Gender(female)^a^	195 (51.5)
Age (years)^b^	9.69 (1.57)
Ethnicity (Caucasian)^a^	340 (89.7)
SES (higher professional)^a^	294 (77.6)
Primary diagnosis^a^	
GAD	107 (28.2)
SAD	96 (25.3)
SoAD	82 (21.6)
Specific phobias	60 (15.8)
Agoraphobia (without panic disorder)	15 (4)
Panic Disorder	6 (1.6)
Secondary diagnoses^a^	
SoAD	168 (44.3)
GAD	140 (36.9)
SAD	124 (32.7)
ODD	78 (20.6)
ADHD	58 (15.3)
MDD	30 (7.9)
Dysthymia	23 (6.1)
Severity measures^a^	
CSR of primary anxiety disorder	5.63 (0.79)
SCAS-C	39.6 (18.75)
SCAS-P	39.93 (15.63)

Data reported:

a n (% of sample).

b Mean (SD).

### 2.2 Measures

#### 2.2.1 Diagnoses

Anxiety disorders and other common comorbid diagnoses were determined using the ADIS-c/p (Silverman et al., 1996); a structured diagnostic interview based on DSM-IV criteria (Silverman et al., 2001). Diagnoses were given alongside a clinical severity rating (CSR) of 4 (moderate psychopathology) or more, based on parent or child report, where CSR's range from 0 (complete absence of psychopathology) to 8 (severe psychopathology). ADIS-c/p assessments were conducted by psychology graduates trained to achieve inter-rater reliability of at least 0.85 for diagnoses and CSRs with an experienced diagnostician (a consultant clinical psychologist). After inter-rater reliability had been achieved assessors were required to discuss one in six subsequent interviews to prevent rater drift. Overall reliability was high for presence or absence of diagnosis (kappa = 0.98) and for the CSR (Intra-class correlation = 0.99).

#### 2.2.2 Anxiety symptoms

Child and parent reported anxiety symptoms were assessed with the Spence Children's Anxiety Scale (SCAS-c/p; Nauta et al., 2004; Spence, 1998). Both the child and parent report versions include 38 items (accompanied by 6 filler items in the child-report version) to rate how often the child experiences each symptom from 0 (never) to 3 (always). Elevated anxiety is represented by total scores above 40 in boys and 50 in girls. Internal consistency for the current sample was good for child (*α* = .89) and parent report (*α* = .89)

#### 2.2.3 Interpretation of ambiguity

Interpretation of hypothetical, ambiguous situations was assessed using an adapted version of the Ambiguous Scenarios Questionnaire (ASQ; Barrett et al., 1996; Creswell and O'Connor, 2006). The questionnaire presents 12 hypothetical situations (six social, e.g., ‘You arrange to have a party at 4 o’clock and by half past 4 no one has arrived’; six non-social, e.g., ‘You are lying in bed at night when you hear a big crash in the night’) and children (a) rate how they would feel in this situation (0 = not at all upset; 10 = very upset; expected negative emotion), (b) give a free response to the question ‘Why do you think this is happening?’ (Threat free response), (c) rate how much they would be able to do about this situation (0 = nothing, 10 = a lot; perceived control), (d) choose which of two alternatives (threat/non-threat, counterbalanced across the 12 situations) they would be more likely to think in this situation (threat forced choice), and (e) report what they would do (avoidance free report).

A psychology postgraduate who was blind to participant characteristics coded all free choice responses. Threat free responses were coded as ‘Threat’ (e.g. ‘Nobody wants to come to my party’) or ‘Non-threat’ (e.g. ‘They must be in a traffic jam’). A second independent coder (an undergraduate psychology student) coded a sub-sample of responses (*n* = 30). Inter-rater reliability was established with good intra-class correlations (ICC = .91 (threat); ICC = .75 (avoidance)). Scores were totaled across situations for each domain (distress, threat (free report), control, threat (forced choice)). Free and forced choice threat scores (*r* = .55, *p* < .001) were combined to reduce the number of variables. Internal consistency for each scale was acceptable (negative emotions *α* = 0.84; threat *α* = 0.59; control *α* = 0.82). Internal consistencies for threat scores were most likely lower as the scales comprise dichotomous variables.

#### 2.2.4 Physiological arousal

Cardiovascular activity during and after a socially relevant stressor task (a presentation performed standing) was used as a measure of physiological arousal. Activity was measured using Actiheart monitors and software (Cambridge Neurotechnology, Cambridge, UK). Two standard ECG electrodes were attached to the child's chest; one just below the sternum and the other towards the left side of the chest. Actiheart calculates average HR (beats per minute, BPM) in 15 s epochs using the number of R waves. In order to ensure that there were no artefacts in the time series used to calculate HR, we used the semi-automated editing software in the Actiheart software to detect and correct artefacts in the inter-beat interval (IBI) time series and visually inspected the time series for any additional artefacts (two independent coders; interrater reliability Kappa >.8).

#### 2.2.5 Social communication deficits

Social communication was assessed using the lifetime version of the Social Communication Questionnaire (SCQ; Berument et al., 1999); a parent report measure based on the Autism Diagnostic Interview-Revised (ADI-R). In keeping with the study rationale, we used the 21 items which have been found to fit well within the Reciprocal Social Interaction (RSI; 13 items, e.g. offering to share or comfort, interest in children and social smiling) and Communication (C; 8 items, e.g. conversation, inappropriate questions and nodding or shaking the head to mean “yes” or “no”) domains (Berument et al., 1999). Parents responded “yes” or “no” to items assessing behaviors occurring at any time (6 items; 1 to assess RSI, 5 to assess C) and behaviors between the age of 4 and 5 years (15 items; 12 to assess RSI, 3 to assess C). Internal consistency was good for the combined RSI and C subscales (RSI-C; *α* = .82).

### 2.3 Ethical considerations

Both the University of Reading and Berkshire NHS research ethics committees approved this study. Potential participants and their parents received written information and had the opportunity to discuss the study with the research team before taking part. Both written consent from primary caregivers and assent from participating children were provided. Both were fully debriefed upon completion of the testing session.

### 2.4 Procedure

Diagnostic interviews and symptom questionnaires were administered to participants and their parents either in clinic rooms within the university or in local satellite clinics. Participants were then invited into the University to complete the interpretation and HR measures. Children and their parents were first given 5-min to play a familiar game to become accustomed to the lab. Children then completed the ASQ with a research assistant. Children and their parent, sat to watch a 5-min DVD (heartrate baseline) before being informed that the child would have 5-min to prepare (with parental support) for a 3-min speech to the researcher and a camera on a topic from a given list (e.g. “My family”). Following the speech, children rated how scared they felt during the task on a scale from 0 (not scared at all) to 10 (very scared). Children and their parents then sat to watch the DVD for a further 5-min (recovery).

### 2.5 Data analysis

Latent Profile Analysis (LPA; carried out with Mplus, Version 7.11 with Combination add-on) was used to investigate the presence of subgroups of children with anxiety disorders. This is a “person-centered” form of cluster analysis that estimates the probability of participants’ membership to a class based on several indicator variables. Here, indicator variables related to the putative maintenance mechanisms for childhood anxiety disorders that are targeted in general forms of CBT (Table 2). The number of indicator variables were reduced^1^ and, as a result, negative interpretations and expected negative emotions were standardized and summed.

**Table 2:** LPA input variables.

Measure.	Variable from measure.	LPA input variable.
ASQ (Cognitive)	Combined threat interpretation.	Negative Interpretation (NI; *r* = .55)
	Expected negative emotions.	Negative Interpretation (NI; *r* = .55)
	Expected Avoidance.	Avoidance.
	Expected Control.	Control.
SCQ (Social Communication Deficits)	Social subscale (RSI)	RSI-C.
	Communication subscale (C)	RSI-C.
Presentation task (Physiological)	Heart rate recovery. (Difference between average BPM during and post social stressor task.)	HR.

Multiple models, with increasing numbers of latent classes, were tested to identify the best latent class solution (Table 3). Various fit indices were used to determine the number of classes that fit the data best. First, the sample size adjusted Bayesian Information Criterion (BIC) and Akaike Information Criterion (AIC) were used; where lower numbers represent a better fit of one model compared to another. Second, the proportion of the sample in each class was required to be more than 5%. Third, the average probabilities for most likely class membership were considered; with acceptable probabilities being more than .7 for a participant belonging in the class in which they are placed or less than .3 for belonging in other classes (Nagin, 2005). Finally, the interpretability of the classes was also taken into account. After determining the number of latent classes, ANCOVAs were used to compare indicator variable means between latent classes, with gender and age as covariates. A Bonferroni correction was applied to account for multiple analyses. Significant main effects were explored with Scheffe's post hoc comparisons (carried out on the unstandardized residuals of each variables having taken age and gender into account). The classes were compared on the presence of clinical characteristics that have been commonly associated with treatment outcome (i.e. SES and the presence of particular anxiety (GAD, SAD and SoAD; the most prevalent disorders in the current sample), mood and behavioral disorders) using Chi-Squared tests. Given that symptom severity is the most consistent predictor of treatment outcome, we conducted sensitivity analyses controlling for baseline anxiety severity (SCAS-c and p totals) in the latent profile analysis. Furthermore, given that there were three items in the SCQ that could feasibly refer to symptoms of social anxiety, sensitivity analyses were also conducted separately, omitting these items. The number of classes and pattern of differences between classes on input variable means was consistent with the original analyses that did not control for anxiety severity or overlapping questionnaire items. Therefore, the results of the original analyses are presented here.

**Table 3:** Latent profile analysis model fits and proportions.

Model.	Fit indices.	*n* and proportion by Class.			
		*Class 1*	*Class 2*	*Class 3*	*Class 4*
*1 Class*	BIC = 9394.48	*N* = 379			
	AIC = 9355.10	100%			
*2 Classes*	BIC = 9336.85	*n* = 329	*n* = 50		
	AIC = 9273.85	86.81%	13.19%		
*3 Classes*	BIC = 9328.74	*n* = 303	*n* = 44	*n* = 32	
	AIC = 9242.11	79.95%	11.61%	8.44%	
*4 Classes*	BIC = 9289.95	*n* = 287	*n* = 45	*n* = 2	*n* = 45
	AIC = 9179.70	75.73%	11.87%	0.53%	11.87%
Average probabilities for membership in each class of the accepted model.	*Class 1*	0.92	0.03	0.05	
	*Class 2*	0.13	0.85	0.02	
	*Class 3*	0.16	0.06	0.78	

Missing data was mostly caused by refusal to take part in particular tasks, limited time for completing all tasks, or (in the case of heart rate measures) clean data not being extractable. We applied the full information maximum likelihood method to deal with missing data (Enders, 2010).

## 3 Results

**Fig. 1:**
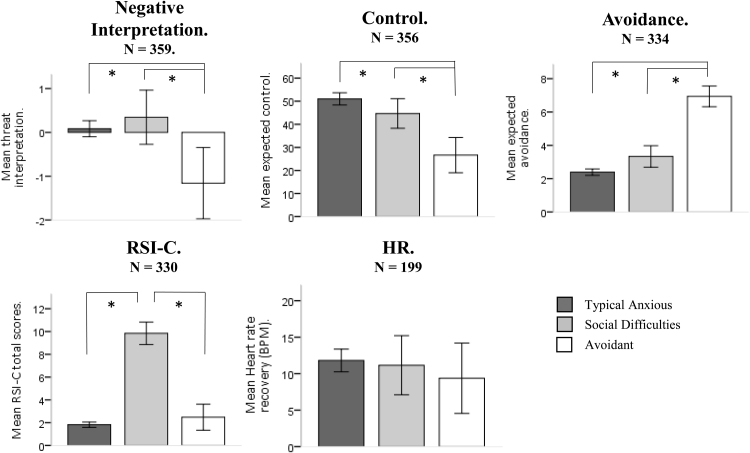
Inter-class differences for LPA input variables within each group. Error bars show 95% confidence intervals. “*” indicates significant differences of p< .05.
(ii) **Do these subgroups align with existing diagnostic categories for anxiety disorders in children?**
  There was a significant main effect of group (Fig. 2) for the proportion of children with any diagnosis (primary or other) of SoAD (χ^2^ (2) = 15.69, p <.001, V = .20) and GAD (χ^2^ (2) = 5.85, p = .05, V = .12), but no significant difference for SAD (χ^2^ (2) = 1.71, p = .43, V = .07). Post hoc tests revealed that the Social difficulties group contained a higher proportion of children with any diagnosis of both SoAD (90.70%) and GAD (81.40%) when compared to the Typical group (SoAD 61.40%, χ^2^ (1) = 14.23, p < .001, ϕ = .20; GAD 62.7%, χ^2^ (1) = 5.78, p = .02, ϕ = .13). The Avoidant group did not differ significantly from the Typical (SoAD, χ^2^ (1) = 2.29, p = .13, ϕ = −.08; GAD, χ^2^ (1) = .001, p = .98, ϕ = .001) nor Social difficulties group (SoAD 75%, χ^2^ (1) = 3.36, p = .07, ϕ = .21; GAD 62.50%, χ^2^ (1) = 3.35, p = .07, ϕ = .21).

(i) **Are there distinct subgroups of clinically anxious children that differ in their expression of the core maintenance mechanisms that are targeted in CBT?**
  Results from the LPA indicated that the three-class model fit the data best. BIC and AIC reduced between one, two, three and four class models (Table 3). However, one of the classes in the four class model did not retain a sufficient proportion of the sample (0.53%). Additionally, average latent class probabilities (Table 3) and the entropy value (.77) for the three-class model were acceptable.
  Although the two-class model also fitted the data well, further investigation, using between group tests, indicated that the three-class model was an elaboration of the two-class model; where the third class was interpretable in and of itself and made theoretic sense. As such, the three-class model was chosen as the most appropriate fit for this data. For ease of interpretation, these groups will hence forth be referred to as the “Typical anxiety”, “Social difficulties”, and “Avoidant” groups.
  A significant main effect of group was found for all input variables except HR recovery (NI, *F*(2, 354) = 7.97, *p* <.001,^2^ *ƞ*^*2*^ = .04; Control, *F*(2, 351) = 18.38, *p* < .001, *ƞ*^*2*^ = .09; Avoidance, *F*(2, 329) = 105.98, *p* < .001, *ƞ*^*2*^ = .39; RSI-C, *F*(2, 325) = 246.23, *p* < .001, *ƞ*^*2*^ = .6; HR, *F*(2, 194) = .82, *p* = .44, *ƞ*^*2*^ = .01; Fig. 1). Post-hoc comparisons revealed that the avoidant group made significantly fewer negative interpretations (*M* = −1.16, *SD* = 2.26) and expected less control (*M* = 26.66, *SD* = 21.19) than both the Typical (NI, *M* = .08, *SD* = 1.56, *p* < .01. *d* = .57; control, *M* = 51.01, *SD* = 22.72, *p* < .001, *d* = 1.12) and the Social difficulties groups (NI, *M* = .35, *SD* = 1.96, *p* < .01, *d* = .76; control, *M* = 44.68, *SD* = 20.02, *p* < .01, *d* = .91) who did not significantly differ from one another (NI, *p* = .26, *d* = .27; control, *p* = .29, *d* = .28). The Avoidant group (*M* = 6.94, *SD* = 1.72) also reported significantly higher avoidance than both the Typical (*M* = 2.39, *SD* = 1.57; *p* < .001, *d* = 1.12) and Social difficulties groups (*M* = 3.33, *SD* = 1.91; *p* < .001, *d* = .91), who did not differ significantly from one another (*p* = .07, *d* = .28). The Social difficulties group (*M* = 9.84, *SD* = 3.23) had significantly higher scores for RSI-C (indicating more difficulties) than both the Typical (*M* = 1.82, *SD* = 1.87; *p* < .001, *d* = 2.85) and avoidant group (*M* = 2.48, *SD* = 2.76; *p* <. 001, *d* = 2.37), who did not differ significantly from one another (*p* = .69, *d* = .17). Here, all significant results demonstrated large effect sizes.

**Fig. 2:**
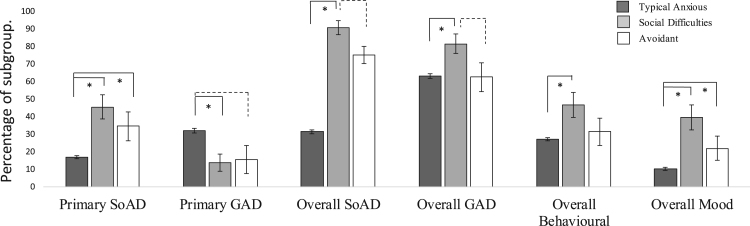
Inter-class differences for the proportion (%) of children with each diagnosis in each group. Error bars show 95% confidence intervals. “*” indicates significant differences of p< .05. Broken lines between groups indicate significance values of 0.07 >p> 0.05.
  A significant main effect of group was also found for the proportion of children with a primary diagnosis of both SoAD (χ^2^ (2) = 21.91, p <.001, V = .24) and GAD (χ^2^ (2) = 8.92, p = .01, V = .15), but not of SAD (χ^2^ (2) = .23, p = .89, V = .03). Post-hoc tests revealed that the Social difficulties (45.50%) and Avoidant groups (34.40%) had a significantly higher proportion of primary SoAD than the typical group (16.8%; χ^2^ (1) = 19.34, p < .001, ϕ = .24; χ^2^ (1) = 5.91, p = .02, ϕ = −.13; respectively), but did not differ significantly from one another (χ^2^ (1) = .94, p = .33, ϕ = .11). Conversely, the Typical group had a significantly higher proportion of children with a primary diagnosis of GAD (31.70%) than the Social difficulties group (13.60%; χ^2^ (1) = 6.03, p = .01, ϕ = −.13). However, the Avoidant group did not significantly differ from the Typical group (15.60%; χ^2^ (1) = 3.54, p = .06, ϕ = .10) or the Social difficulties group (χ^2^ (1) = .06, p = .81, ϕ = −.03).
(iii) **Do these subgroups differ on clinical characteristics that commonly predict treatment outcome?**
  There was a significant main effect of group for age and gender (F(2, 375) = 4.50, p = .01, ƞ^2^ = .02; χ^2^(2) = 9.68, p = .0, V = .16; Fig. 3). Post hoc comparisons revealed that the Social difficulties group were significantly older (M = 10.27, SD = 1.45) than the Typical group (M = 9.57, SD = 1.57; p = .02, d = .46) but not the Avoidant group (M = 9.97, SD = 1.53; p = .70, d = .20), who did not differ significantly from the Typical group (p = .40, d = .46). There were also significantly higher proportions of males in the Social difficulties (63.6%) and Avoidant groups (65.6%) compared to the Typical group (44.60%; χ^2^ (1) = 5.42, p = .02, ϕ = −.13; χ^2^ (1) = 5.16, p = .02, ϕ = .12; respectively), with the Social difficulties and Avoidant groups not differing significantly from each other (χ^2^ (1) = .03, p = .86, ϕ = .02).

**Fig. 3:**
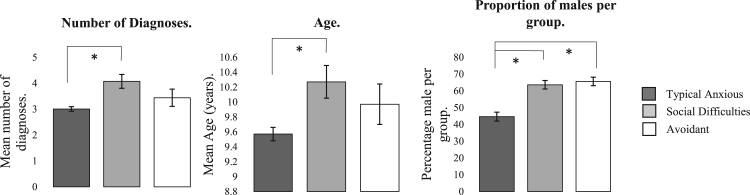
Inter-class differences for demographic variables within each group. Error bars show 95% confidence intervals. “*” indicates significant differences of p< .05.
  There was a significant main effect of group for children with a secondary diagnosis of a mood *(χ*^*2*^ (2) = 28.65*, p* < .001, V = .28) or behavioral disorder (*χ*^2^ (2) = 6.88, *p* = .03, *V* = .14; Fig. 3). Post-hoc tests revealed significantly higher proportions of children with a comorbid diagnosis of behavioral (46.50%) and mood disorders (39.50%) in the Social difficulties compared to the Typical group (behavioral: 27.10%, *χ*^2^ (1) = 6.85, *p* = .01, *ϕ =* .14; mood: 9.90%, *χ*^2^ (1) = 28.17, *p* < .001, ϕ = .29). The Avoidant group also had a significantly higher proportion of children with a co-morbid diagnosis of a mood disorder (21.90%) compared to the Typical group (*χ*^2^ (1) = 4.22, *p* = .04, *ϕ =* −.11), but all other group differences were not significant (Behavioral: Social difficulties and Avoidant (31.30%), *χ*^2^ (1) = 1.78, *p* = .18, *ϕ* = .15; Typical and Avoidant, *χ*^2^ (1) = .26, *p* = .61, *ϕ* = −.03; Mood: Social difficulties and Avoidant, *χ*^2^ (1) = 2.63, *p* = .11, *ϕ =* .19).
  Finally, there was a significant main effect of group for the number of comorbid disorders diagnosed (*F*(2, 373) = 8.80, *p* <.001, ƞ^*2*^ = .04). Post hoc comparisons revealed that children in the Social difficulties group had significantly more comorbid diagnoses (Fig. 2; *M* = 4.07, *SD* = 1.76) than in the Typical (*M* = 3.00, *SD* = 1.54; *p* <.001, d = .63), but not Avoidant group (*M* = 3.44, *SD* = 1.88; *p* = .28, *d* = .33) who also did not differ significantly from the Typical group (*p* = .34, *d* = .25).

## 4 Discussion

This study explored the presence of subgroups of clinically anxious children for whom the putative mechanisms that are commonly targeted in general CBT for child anxiety disorders may apply to different degrees. It also evaluated whether these subgroups were associated with traditional diagnostic categories and with clinical characteristics that predict CBT outcomes. Three latent classes were identified which were characterized as follows: the “typical anxiety group” contained most of the sample (79.95%) and the highest proportion of children with a GAD diagnosis (31.70%). In contrast, the “social difficulties” group had high parent rated social and communication difficulties compared to both other groups. This group had a higher proportion of males (63.30%) which may not be surprising given the higher prevalence of social communication difficulties among males compared to females (Fombonne, 2005). The “social difficulties” group were also older in age and had the highest proportion of children with a primary diagnosis of SoAD (45.50%). As we do not know the age of ‘onset’ of the child's difficulties, we cannot conclude whether these sorts of difficulties emerge later or whether families seek, or at least access help for these sorts of difficulties later. However the findings are certainly consistent with findings that individuals with social anxiety disorder have particularly long delays between the onset of difficulties and help seeking compared to those with, for example, generalized anxiety disorder (Wang et al., 2005). Notably, children in the “social difficulties” group had more co-morbid disorders than the other groups, yet it remained a distinct group after severity was controlled for. Finally, the “avoidant” group reported high avoidance and low perceived control. It is interesting to note that the ‘avoidant’ group also reported low levels of negative interpretation and negative emotional responses. It is unclear whether this reflects a tendency to avoid thinking about negative outcomes, or a general tendency for avoidance even in low risk situations; potentially reflecting a general tendency to avoid uncertainty.

Although the subgroups differed on many of the input variables, there were no significant differences between the groups for HR recovery from a presentation task. This may suggest that all anxious children display comparable levels of physiological arousal. However, the sample size was significantly reduced for this variable due to missing data. As such, the analysis was under powered and we are, therefore, unable to confidently draw conclusions from this result.

The current findings may go some way to explaining the inconsistent findings of previous research in to mechanisms that maintain childhood anxiety disorders by identifying subgroups of clinically anxious children who express these mechanisms to varying degrees. Notably, these subgroups did not align neatly with existing diagnostic categories: although, there were associations between some latent classes and diagnostic categories (e.g. GAD in the Typical group and SoAD in the Social difficulties group), with small to medium effect sizes. For example, although the vast majority of children in the ‘social difficulties’ group had a diagnosis of SoAD (primary or otherwise; 92%), a small proportion did not (8%). Furthermore, only 15.7% of children with a SoAD diagnosis (primary or otherwise) were in the social difficulties group, with 74.7% in the typical group and 9.6% in the avoidant group. These findings suggest that treatments targeting social communication difficulties may benefit some, but not all, children with a SoAD diagnosis. Furthermore, some children with other anxiety disorders (not just SoAD) may also benefit from treatments that target social communication difficulties; approximately 15% of the children with diagnoses of both SAD and GAD were in the “social difficulties” group. Similar proportions of children with SAD and GAD were also classified in the “typical” and “avoidant” groups. These findings suggest that the traditional diagnostic categories may not best tell us which maintenance mechanisms need to be targeted in treatment.

The data driven identification of these subgroups has potential implications for delivery of more targeted treatments that could be more effective and efficient. Indeed, in adult populations, treatments that monitor and target specific maintenance factors have been shown to outperform many other types of treatment, including general forms of psychotherapies (e.g. Cognitive Therapy (CT) for SoAD; Clark et al., 2006).

### 4.1 Limitations

This study has notable strengths including the inclusion of a relatively large clinical sample and a range of methods to address cognitive, physiological and social domains. However, several limitations should be highlighted. For example, the measure of physiological arousal was limited to heart rate recovery. This was primarily because previous studies have shown slower HR recovery in anxious children after a social stressor and have failed to show differences in HR reactivity (Schmitz et al., 2011, Alkozei et al., 2015). However, findings could have been confounded by differences in state anxiety (Alkozei et al., 2015), excessive movement (e.g., fidgeting in anxious children), body mass index, medical history or exercise patterns. It is also important to note that participants sat for one part of the task and stood for another, limiting the interpretation of the within group repeated measures. These confounds may have contributed to the null results found between groups on HR recovery.

We included a widely used child self-report measure of interpretation of ambiguity in which children are presented with hypothetical scenarios, however the ecological validity of this measure is yet to be established. Our measure of children's social communication difficulties is also widely used and well validated, with items which are clearly distinct from measures of social anxiety. However, the measure relies on subjective parent report and recall.

Sample characteristics that may limit the generalizability of the findings also need to be highlighted. First, this was a treatment seeking sample with relatively high SES. Second, given that differences have been found in the association between anxiety and interpretation in preadolescent and adolescent children (Waite et al., 2015), we restricted the age range to 7–12 year old's so further studies with adolescents are required. Finally, we focused on a restricted range of putative mechanisms of anxiety and characteristics that are associated with CBT outcomes; further studies are required which consider broader, relevant variables such as parental anxiety and parenting styles (Compton et al., 2014).

## 5 Conclusions

These limitations notwithstanding, the results from this study suggest that there are subgroups of clinically anxious children who differ in the extent to which they express the putative maintenance mechanisms that are targeted in traditional CBT approaches. Further studies are now required to establish whether treatments that target specific mechanisms among particular subgroups of children will lead to more effective and efficient treatments.

## Footnotes

1 The LPA was also run with the individual variables, showing the same pattern of results as analyses using the combined variables.

2 No significant differences were found between groups for threat responses to social and non-social scenarios when analysed separately.
